# Supplementary material for: Venomix: a simple bioinformatic pipeline for identifying and characterizing toxin gene candidates from transcriptomic data
Source: PeerJ. 2018 Jul 31;6:e5361. doi: 10.7717/peerj.5361 (PMC6074769; doi:10.7717/peerj.5361)
Supplement: Supplemental Information 3 [file peerj-06-5361-s003.gz › FinalOutput_E-20/Snaclec_echicetin_subunit_alpha_1/finaltree.pdf]

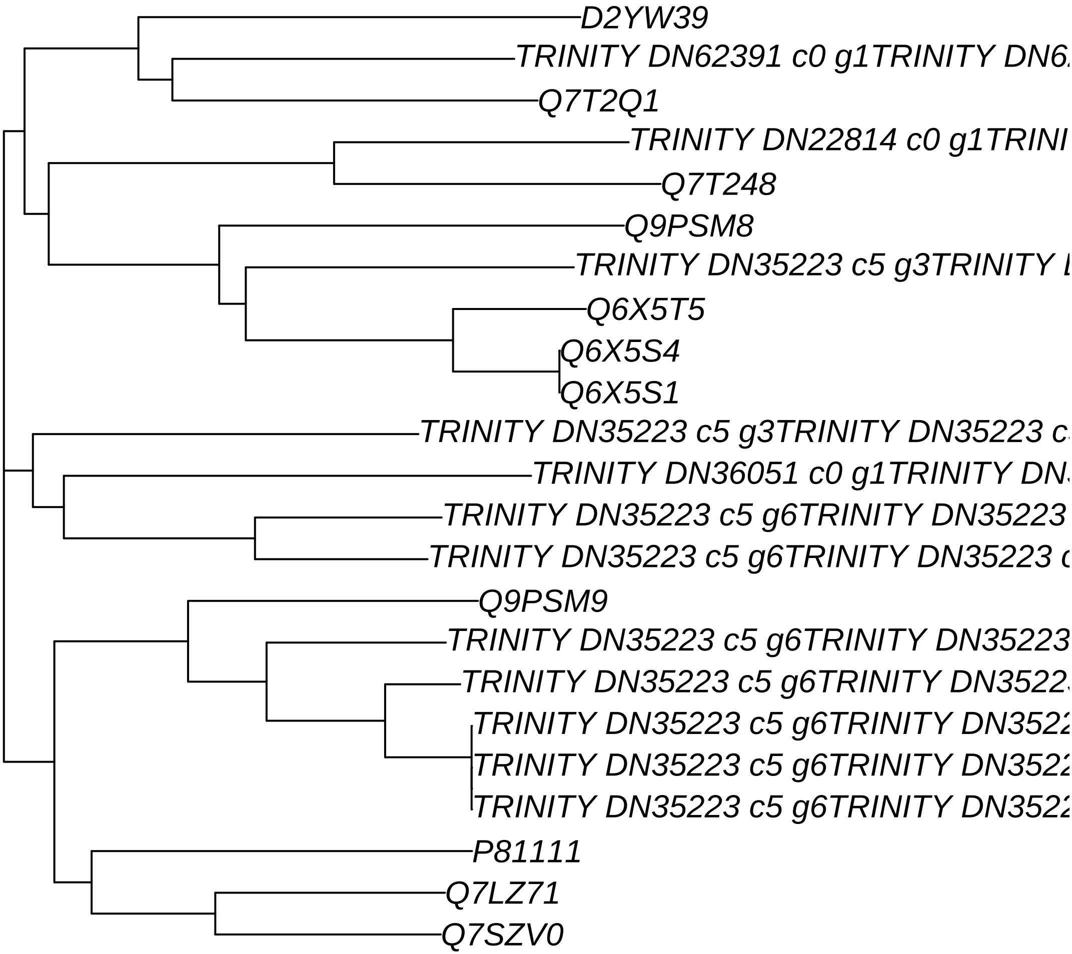

D2YW39
TRINITY DN62391 c0 g1TRINITY DN6
Q7T2Q1
TRINITY DN22814 c0 g1TRINI
Q7T248
Q9PSM8
TRINITY DN35223 c5 g3TRINITY
Q6X5T5
Q6X5S4
Q6X5S1
TRINITY DN35223 c5 g3TRINITY DN35223 c
TRINITY DN36051 c0 g1TRINITY DN
TRINITY DN35223 c5 g6TRINITY DN35223
TRINITY DN35223 c5 g6TRINITY DN35223
Q9PSM9
TRINITY DN35223 c5 g6TRINITY DN35223
TRINITY DN35223 c5 g6TRINITY DN3522
TRINITY DN35223 c5 g6TRINITY DN3522
TRINITY DN35223 c5 g6TRINITY DN3522
TRINITY DN35223 c5 g6TRINITY DN3522
P81111
Q7LZ71
Q7SZV0
